# A simplified and scalable synthesis of artesunate

**DOI:** 10.1007/s00706-016-1865-9

**Published:** 2016-12-02

**Authors:** Armin Presser, Andrea Feichtinger, Silke Buzzi

**Affiliations:** Institute of Pharmaceutical Sciences, University of Graz, Graz, Austria

**Keywords:** Antimalarial, Peroxides, Natural products, Reductions, Green chemistry

## Abstract

**Abstract:**

An efficient and economically viable approach for the large-scale conversion of artemisinin into the antimalarial frontline drug artesunate was developed. This advanced synthesis includes an NaBH_4_-induced reduction, followed by an esterification with succinic anhydride under basic conditions. The entire conversion follows the principles of green chemistry, i.e., application of reusable solvents.

**Graphical abstract:**

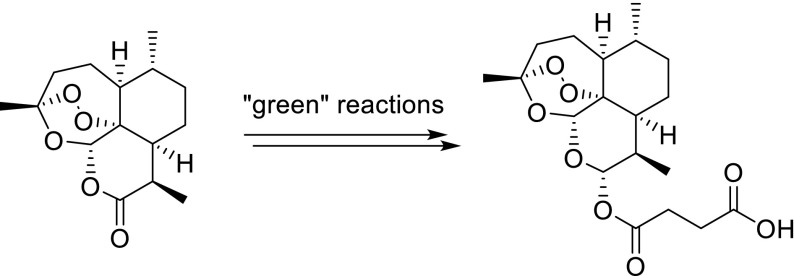

## Introduction

Malaria, caused by parasitic protozoans of the *Plasmodium* type, still remains a life-threatening disease with about 600,000 death cases each year, with many of them being children under 5 years. The sesquiterpene endoperoxide artemisinin (**1**) [1, 2], is the lead component of the so-called artemisinin combination therapies (ACTs), which is currently the most valuable weapon in the fight against this disease. Several easily accessible artemisinin derivatives, including dihydroartemisinin (DHA, **2**) and artesunate (**3**), exhibit potent antimalarial activity against drug-resistant malaria strains [3–5]. In addition, they also show remarkable activities against other parasites and various cancer types [4, 6].

Artemisinin was discovered in the 1970s as a result of an extensive screening of Chinese herbal extracts in the search of new antimalarial agents [7]. Currently, the primary method for the production of artemisinin is the isolation from dried leaves of the plant *Artemisia annua*. Shortcomings of existing processes are poor yields and environmental pollution [8]. Furthermore, the global supply of this life-saving drug exclusively from natural sources remains highly limited. As a result, several novel approaches for large-scale production of artemisinin have been developed [9–13].

ACT medications rely not only on the procurement of artemisinin, also the derivatives DHA (**2**, also known under its INN identification artenimol) and artesunate (**3**) became frontline drugs due to stronger activity and improved bioavailability compared to the parent compound [1, 14]. The transformation of artemisinin into artesunate was commonly realized by a two-step process involving the reduction with NaBH_4_ [15–19], KBH_4_ [20, 21] or diisobutylaluminium hydride (DIBAL) [22, 23], followed by esterification using succinic anhydride [24, 25]. However, these syntheses described in the scientific literature are not suitable for an industrial large-scale process because they require very low temperature, highly expensive or toxic reagents and solvents or provide only moderate yields. Recently, some continuous flow protocols for the preparation of **2** and **3** were published [26–28]. Flow protocols have some technical advantages over batch protocols. However, batch processing still dominates the manufacturing of pharmaceuticals today.

On the basis of our earlier studies sponsored by Medicines for Malaria Ventures (MMV) [29], we report herein a facile and scalable synthetic route for the preparation of dihydroartemisinin (**2**) and artesunate (**3**) from **1** in high yields following the principles of green chemistry, i.e., the minimization of waste, avoidance of toxic solvents, and elimination of unnecessary steps.

### Results and discussion

Our initial work started with the well-described reduction of artemisinin with NaBH_4_ in methanol. THF or dioxane were also used as solvents [30]; however, because of ecological and economic reasons, the use of these solvents was ruled out [31–33]. DIBAL in CH_2_Cl_**2**_ seems to be a superior reagent for the conversion of artemisinin to DHA [23], but it is more expensive and requires extremely low temperature. The also known procedure with KBH_4_ in the presence of a phase-transfer catalyst [20] shows some inconsistencies and contains a rather hazy experimental part.

After assessing diverse instructions from the literature, we determined the most suitable conditions for the reduction of artemisinin: first, we identified the amount of NaBH_4_ required for the complete transformation. As a consequence of the competing methanol-induced solvolysis, a certain excess of the reagent is definitely required. It is known that the instability of NaBH_4_ in MeOH can be overcome by the addition of a base [34], but the extreme vulnerability of DHA towards small amounts of strong bases [21] excludes this approach. Apparently, the application of a lower reaction temperature leads to a slower decomposition of NaBH_4_ by MeOH [21]. As a consequence, the reaction time has to be kept short, and the reaction temperature kept as low as possible. Due to the decrease of the reaction temperature, the excess of NaBH_4_ could be considerably reduced as compared to the published instructions. However, a contamination of the reagent with traces of strong bases will also have a negative impact on the yield.

**Figure:**
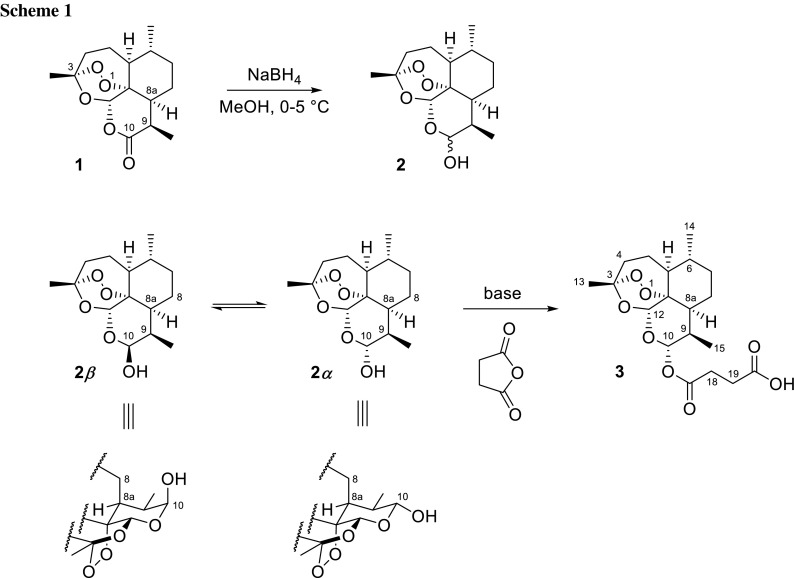

The best results were achieved when 2.5 equiv. of NaBH_4_ were added to a suspension of approx. 0.6 M artemisinin in MeOH at 0–5 °C over 30 min and a total reaction time of 1 h (Scheme 1). Two different types of NaBH_4_, powder and granulate, were tested. Even though the yields are identical, the use of granulated material is preferred because the production of toxic dust during handling is minimized. Subsequently, the work-up conditions were optimized. After destroying the excess of NaBH_4_ with acetic or hydrochloric acid, DHA is usually precipitated by the addition of water. Through this procedure, DHA could be obtained in satisfactory yields (79–89%) as published previously [16, 19, 21]. In an alternative strategy, the yield of DHA could be further enhanced by neutralization with 30% acetic acid/MeOH at 0–5 °C, concentration, and multiple extraction of the white residue using Et-OAc. Evaporation under reduced pressure provided **2** as a white crystalline powder in 98% yield, which can be used in the next step without further purification. To reduce the large consumption of Et-OAc, the solvent was distilled and reused.

**Fig. 1:**
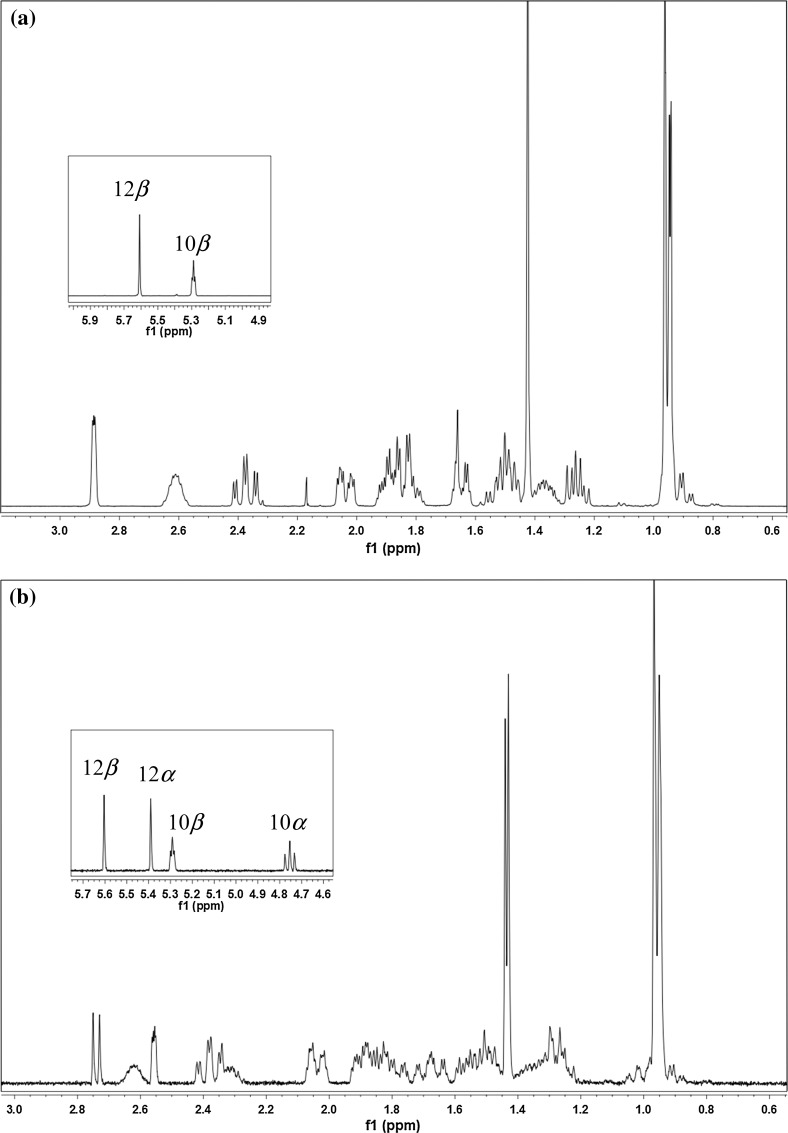
^1^H NMR of DHA (**2**): immediately after dissolution (**a**) and after 12 h (**b**)

Bulk solid DHA results solely as *β*-epimer of the lactol hemiacetal [35]. Addition of CDCl_3_ provides a solution consisting exclusively of **2** ***β***, which equilibrates gradually to a 1:1 mixture of **2** ***α*** and **2** ***β***. The epimerization is clearly indicated by arising signals at 5.39 ppm (H-12α) and 4.75 ppm (H-10α) in the ^1^H NMR spectrum (see Fig. 1). The equilibrium ratio is depending on the solvent [36]: in CH_3_CN/H_2_O which we used for HPLC analysis, the relation of **2** ***α*** and **2** ***β*** is 3:1.

Acylation of DHA (**2**) to artesunate (**3**) was first realized with succinic anhydride in the presence of pyridine as a base/solvent [24]. The use of catalytic DMAP was also reported [37] with a yield of 65%. Aside from Chinese publications, there exist only marginal instructions [25] for the preparation of **3** so far. Also, a patent specification with Et_3_N as base in acetone, THF, or dioxane promising yields up to 97% [38] was not reproducible [36]. Pharmaceutically approved artesunate is an *α*-linked dihydroartemisinin hemisuccinate. It was reported that acylation of DHA in alkaline media led almost exclusively to *α*-configured derivatives [35, 39]. An obvious reason may be the crowded environment of the axial hydroxyl in the *β*-epimer or the higher stability and thermodynamically favored conformation of **2** ***α***. The classical esterification method using acid chlorides or anhydrides in pyridine (Einhorn method) requires anhydrous conditions. In our first attempt, we replaced the originally used pyridine by Et_3_N and chose Et-OAc as a reusable and ecologically more acceptable solvent. A supplemental addition of DMAP as well as the usage of anhydrous solvents appeared to be negligible. To further improve the reaction conditions, we investigated the stoichiometric ratio of the reagents. As shown in Table 1, the esterification occurred almost quantitatively with a 1.4-fold excess of succinic anhydride using 0.6 equiv. of Et_3_N. The reaction can take place at RT or even higher temperature (up to 50 °C) without any difference in yield.

**Table 1:** Synthesis of artesunate with different stoichiometric ratios and temperatures

Entry	Succ. anhydr./equiv	Et_3_N/equiv	Temp./°C	Yield/%^a^
1	2.0	1.1	25	86
2	1.5	1.1	25	81
3	1.5	1.1	50	80
4	1.2	1.1	25	58
5	1.5	0.8	25	85
6	1.3	0.8	25	83
7	1.3	0.6	25	89
8	1.3	0.6	40	88
9	1.4	0.6	25	94

Reaction conditions: dihydroartemisinin (**2**, 35.2 mmol), succinic anhydride and Et_3_N as stated above, 30 cm^3^ solvent; the reactions were monitored by TLC until the apparent consumption of the limiting substrate

^a^Isolated yield

Unfortunately, the recycling of Et-OAc by distillation leaves a water content up to 8.5% [40] which negatively affects the yield when reused solvent was applied. This difficulty was overcome by changing the reaction medium to isopropyl acetate. Because of the lower moisture content (1.5% determined via Carl Fischer titration), even after recycling of the solvent, artesunate was obtained in the same excellent yields. ^1^H NMR spectroscopic analyses confirmed that exclusively *α*-artesunate was formed; the large trans-diaxial coupling of H-10 at 5.67 ppm (*J*_10,9_ = 9.7 Hz) pinpoints the requested configuration at C-10.

## Conclusion

In summary, we report on the development of an advanced synthetic route for the conversion of the natural product artemisinin into semisynthetic artesunate by NaBH_4_-induced reduction and subsequent esterification under basic conditions as pivotal steps. Using our synthetic approach, feasibility and scalability of the synthesis can be dramatically improved while the overall yield remains among the best so far documented. This procedure should favorably complement the existing routes and may represent an additional entry to the production of artesunate at low costs.

### Experimental

Melting points were obtained on a digital melting point apparatus (Electrothermal IA 9200). The NMR spectra were measured on a Varian Unity Inova 400 instrument (at 298 K) using 5-mm tubes. Chemical shifts were given in parts per million (ppm); the tetramethylsilane (TMS) resonance (0.00 ppm) was used as internal standard. Coupling constants (*J*) were reported in Hertz (Hz). ^1^H and ^13^C resonances were assigned using ^1^H, ^1^H and ^1^H, ^13^C correlation spectra. ^1^H and ^13^C resonances are numbered as given in the formulae. The signals marked with an asterisk are interchangeable. The IR spectra were recorded on an ALPHA FT-IR-spectrometer (Bruker). Optical rotation was determined on a P-2000 polarimeter (Jasco). The water content of the solvents was quantified via Carl Fischer titration on a TitroLine 7500 KF (SI Analytics). HPLC separations were performed on a Merck Hitachi HPLC apparatus D6000A consisting of a pump L6200 and UV–Vis-detector L4250. Diastereomer separation was achieved using a LiChrospher^®^ 100 RP–18 (5 μm, 125 × 3 mm, Merck) HPLC column, operated at 30 °C. Each 10-min chromatographic run was carried out at a flow rate of 0.5 cm^3^/min with an isocratic mobile phase consisting of acetonitrile (20%) and Millipore water (80%). Runtime was 10 min and detection wavelength was 224 nm. The purity of artesunate was checked by HPLC analysis performed on an Agilent 1200 series equipped with an autosampler, a quaternary pump system and a photodiode array detector. An Agilent Zorbax SP-C18 column (particle size 3.5 μm; 2.1 × 150 mm with guard cartridge) was used at a flow rate of 0.30 cm^3^/min. The chromatographic method was performed with a gradient of acetonitrile (A) in Millipore water (B), both with each 0.1% HCOOH, from 10 to 90% A in B within 20 min, then to 100% A within 5 min followed by returning to starting conditions within 1 min and re-equilibration for 8 min. 10 mm^3^ of sample was injected and detection was done at 220 nm. Materials: TLC was carried out on Merck TLC plates (silica gel 60 *F*_254_ 0.2 mm, 200 × 200 mm). TLCs were visualized by spraying with cerium(IV) sulfate/ammonium molybdate and subsequent heating with a heat gun. The solvents were concentrated by rotary evaporation below 50 °C. Purity and homogeneity of compounds were assessed by TLC and HPLC methods.

#### (3*R*,5a*S*,6*R*,8a*S*,9*R*,10*S*,12*R*,12a*R*)-Decahydro-3,6,9-trimethyl-3,12-epoxy-12H-pyrano[4,3-j][1,2]benzodioxepin-10-ol (dihydroartemisinin, ***2***)

Artemisinin (10.0 g, 35.4 mmol) was suspended in 60 cm^3^ MeOH and cooled in an ice bath to 0–5 °C. To the suspension, 3.35 g of NaBH_4_ (88.5 mmol) was added in small portions over a period of 30 min. After the addition of NaBH_4_ was complete, the reaction was allowed to reach ambient temperature and was stirred vigorously under Ar until the TLC showed complete consumption of **1** (~30 min). The mixture was neutralized (pH 5–6) with 30% AcOH/MeOH, concentrated under reduced pressure and finally lyophilized. The white residue was extracted with Et-OAc several times. The combined Et-OAc extracts were filtered and evaporated to dryness to give 9.87 g (98%) DHA as a white crystalline powder, which can be used in the next step without further purification. *R*_f_ = 0.46 (CH_2_Cl_2_:MeOH = 20:1), m.p.: 150–151 °C (Ref. [41], 152–153 °C). The spectroscopic data were found to be identical with the ones described in Refs. [41–43].

#### (3*R*,5a*S*,6*R*,8a*S*,9*R*,10*S*,12*R*,12a*R*)-Decahydro-3,6,9-trimethyl-3,12-epoxy-12H-pyrano[4,3-j][1,2]benzodioxepin-10-ol, hydrogen succinate (artesunate, ***3***)

A solution of 4.93 g succinic anhydride (49.2 mmol) in 30 cm^3^ isopropyl acetate was treated under Ar with 2.94 cm^3^ Et_3_N (21.1 mmol). To this solution, 10.0 g of **2** (35.2 mmol) was successively added over a period of 30 min and the mixture was stirred at ambient temperature for 4 h. Then, it was quenched with H_2_O and H_2_SO_4_ (2 N) until pH 5 was reached and stirred for a few minutes to achieve phase separation. The aqueous phase was extracted thoroughly with isopropyl acetate and the combined organic layers were concentrated under reduced pressure providing almost pure *α*-artesunate (impurities <1%) as fine white needles. Yield 12.7 g (94%).

A batch size over 50 g DHA requires a modified work-up: After quenching with a small amount of H_2_O and neutralization with H_2_SO_4_ (2 N), the mixture was diluted with isopropyl acetate and heated to 50 °C to dissolve precipitated **3**. The organic layer was washed with warmish H_2_O and concentrated under reduced pressure providing *α*-artesunate as described above. *R*_f_ = 0.27 (CH:Et-OAc = 1:1), m.p.: 131–133 °C (MeOH:H_2_O = 1:2) (Ref. [39], 135 °C). The spectroscopic data were found to be identical to the ones described in Refs. [39, 44]. Although the crystal structure of **3** is already known [39], to the best of our knowledge, a complete assignment of the NMR signals has never been published: ^1^H NMR (400 MHz, DMSO-*d*_*6*_): *δ* = 5.67 (d, *J* = 9.7 Hz, 1H, H-10), 5.56 (s, 1H, H-12), 2.63–2.58 (m, 2H, H-18′, H-19′), 2.53–2.48 (m, 2H, H-18″, H-19″), 2.35–2.23 (m, 1H, H-9), 2.18 (td, *J* = 13.9, 3.9 Hz, 1H, H-4′), 2.00 (dt, *J* = 13.9, 3.9 Hz, 1H, H-4″), 1.81 (dq, *J* = 10.3, 3.5 Hz, 1H, H-5′), 1.67–1.61 (m, 1H, H-8′), 1.64–1.57 (m, 1H, H-7′), 1.58–1.51 (m, 1H, H-8a), 1.48–1.41 (m, 1H, H-8″), 1.47–1.37 (m, 1H, H-6), 1.36–1.27 (m, 1H, H-5″), 1.29 (s, 3H, H-13), 1.19 (dd, *J* = 11.3, 6.4 Hz, 1H, H-5a), 1.02–0.88 (m, 1H, H-7″), 0.89 (d, *J* = 6.4 Hz, 3H, H-14), 0.77 (d, *J* = 7.1 Hz, 3H, H-15) ppm.; ^13^C NMR (100 MHz, DMSO-*d*_*6*_): *δ* = 173.7 (C-20), 171.5 (C-17), 104.0 (C-3), 92.2 (C-10), 91.1 (C-12), 80.3 (C-12a), 51.6 (C-5a), 45.0 (C-8a), 36.4 (C-4, C-6), 34.2 (C-7), 32.1 (C-9), 29.2* (C-18), 28.9* (C-19), 26.0 (C-13), 24.7 (C-5), 21.5 (C-8), 20.5 (C-14), 12.2 (C-15) ppm.
